# Evaluation of Quality of Life in Hemodialysis Patients

**DOI:** 10.1192/j.eurpsy.2026.11685

**Published:** 2026-07-31

**Authors:** H. Hsine, S. Kolsi, A. Bouaziz, W. Abbes

**Affiliations:** 1 Psychiatry, Clinic La Metairie, Nyon, Switzerland; 2 Psychiatry, Gabes Hospital, Gabes, Tunisia

## Abstract

**Introduction:**

Chronic kidney disease (CKD) currently represents a major public health challenge worldwide.

Hemodialysis constitutes an essential renal replacement therapy, but it is often associated with a significant impact on patients’ quality of life.

**Objectives:**

To assess the quality of life of hemodialysis patients and to identify the associated factors.

**Methods:**

We conducted a cross-sectional, descriptive, and analytical study at Gabes University Hospital over a four-month period (February 2025 to May 2025). The study population included chronic hemodialysis patients followed at the hospital’s hemodialysis unit.

Data collection was performed using a structured questionnaire and validated psychometric tools. Quality of life was assessed using the SF-12 questionnaire, the short version of the *Medical Outcomes Study Short Form General Health Survey* (SF-36). This instrument evaluates eight domains of quality of life: general and mental health, physical and social functioning, physical and emotional health, pain, and vitality.

The SF-12 includes 12 items, from which physical and mental quality of life scores are calculated using a standardized algorithm, ranging from 0 to 100. Interpretation is based on two main components: the Physical Component Summary (PCS-12) and the Mental Component Summary (MCS-12).

**Results:**

Assessment of quality of life in 33 hemodialysis patients revealed a marked impairment in the physical component (PCS-12). Nearly two-thirds of the patients (66.7%) had a PCS score between 30 and 39, indicating a moderate to severe disability.

Similarly, the evaluation highlighted a decline in psychological well-being (MCS-12).

The mean PCS and MCS scores were 38.29 ± 6.01 and 43.50 ± 9.04, respectively, reflecting an overall deterioration in quality of life.

Low family support was the only significant determinant of a PCS score below 50 (p = 0.027), underscoring its role in the physical dimension of quality of life.

**Conclusions:**

Our study highlighted a considerable impairment in quality of life among hemodialysis patients, generating substantial challenges that affect not only patients themselves but also their families and healthcare providers. These findings emphasize the need for a multidisciplinary approach in the management of individuals undergoing hemodialysis.

**Disclosure of Interest:**

None Declared

